# Accessibility and potency of uterotonic drugs purchased by simulated clients in four districts in India

**DOI:** 10.1186/s12884-014-0386-y

**Published:** 2014-11-13

**Authors:** Cynthia Stanton, Deepak Nitya Nand, Alissa Koski, Ellie Mirzabagi, Steve Brooke, Breanne Grady, Luke C Mullany

**Affiliations:** Department of Population, Family, and Reproductive Health, Johns Hopkins Bloomberg School of Public Health, Baltimore, MD USA; PATH, New Delhi, India; PATH, Seattle, WA USA; Department of International Health, Johns Hopkins Bloomberg School of Public Health, Baltimore, MD USA

**Keywords:** Medicines quality, Uterotonics, Potency, Oxytocin, Methylergometrine, India

## Abstract

**Background:**

Surveillance of drug quality for antibiotics, antiretrovirals, antimalarials and vaccines is better established than surveillance for maternal health drugs in low-income countries, particularly uterotonic drugs for the prevention and treatment of postpartum hemorrhage. The objectives of this study are to: assess private sector accessibility of four drugs used for uterotonic purposes (oxytocin, methylergometrine, misoprostol, valethamate bromide); and to assess potency of oxytocin and methylergometrine ampoules purchased by simulated clients.

**Methods:**

The study was conducted in Hassan and Bagalkot districts in Karnataka state and Agra and Gorakhpur districts in Uttar Pradesh state. A sample of 877 private pharmacies was selected (using a stratified, systematic sampling with random start), among which 847 were successfully visited. The target sample size for assessment of accessibility was 50 pharmacies per drug, per district. The target sample size for potency assessment was 100 purchases each of oxytocin and methylergometrine across all districts. Successful drug purchases varied by state.

**Results:**

In Agra and Gorakhpur, 90%-100% of visits for each of the drugs resulted in a purchase. In Bagalkot and Hassan, only 29%-52% of visits for each drug resulted in a purchase. Regarding potency, the percent of active pharmaceutical ingredient was assessed using United States Pharmacopeia monograph #33 for both drugs; 193 and 188 ampoules of oxytocin and methylergometrine, respectively, were assessed. The percent of oxytocin ampoules outside manufacturer specification ranged from 33%-40% in Karnataka and from 22%-50% in Uttar Pradesh. In Bagalkot and Hassan, 96% and 100% of the methylergometrine ampoules were outside manufacturer specification, respectively. In Agra and Gorakhpur, 54% and 44% were outside manufacturer specification, respectively.

**Conclusion:**

Private sector accessibility of uterotonic drugs in study districts in Karnataka warrants attention. Most importantly, interventions to assure quality oxytocin and particularly methylergometrine are needed in study districts in both states.

## Background

Postpartum hemorrhage (PPH) is the leading cause of maternal death both globally and in India [1,2]. In India, PPH deaths account for 31-38% of all maternal deaths [2]. The World Health Organization (WHO) recommends use of a uterotonic drug for all births for the prevention of PPH, and cites oxytocin as the drug of choice for prevention and treatment of PPH. Alternative uterotonics, such as misoprostol and ergometrine are recommended where oxytocin use is not feasible [3]. Current Government of India guidelines also promote prophylactic use of oxytocin, where feasible, or misoprostol for deliveries attended by auxiliary nurse midwives through medical officers [4,5]. Oxytocin and ergometrine have been included in the WHO Model Essential Medicines List since its inception in 1977; misoprostol was added to the core list in 2005 [6]. More recently, all three drugs appear in the Essential Interventions, Commodities and Guidelines for Reproductive, Maternal, Newborn and Child Health [7]. Oxytocin and misoprostol also figure as two of only 13 drugs prioritized by the United Nations Commission on Life-Saving Commodities for Women and Children which proposes recommendations to address not only limited access to these drugs but also interventions to improve the their quality [8].

The quality of pharmaceutical medicines in general has received increased attention globally from public health professionals over the last decade. This is particularly true in India given the increasingly important role India plays as a drug exporter [9]. Globally, the predominant concern regarding quality of medicines has been counterfeit drugs [10,11]. However, the issue of substandard and/or degraded drugs is also being recognized as a major public health concern [12,13]. Given that distinct solutions are required for each of these problems, Newton and colleagues have stressed the need for a consensus on definitions and an end to interchangeable use of these terms [14]. Globally and in India, attention has focused primarily on anti-infective drugs such as antiretroviral drugs, antibiotics, anti-tuberculosis drugs and anti-malarials [12,15] Manufacturing to specific standards and monitoring of product quality of such drugs is mandated as a prerequisite for funding from international programs such as the Global Fund to Fight AIDS, Tuberculosis and Malaria [16]. Regarding drug degradation, vaccine management is a domain that has been highly promoted and supported for several decades by the WHO-initiated Expanded Program for Immunization, with an emphasis on cold chain maintenance, equipment, training and monitoring [17]. Consequently, for health care planners in infectious disease, awareness of drug quality maintenance issues has been a high priority for years.

The same is not true for health care planners in maternal health. For example, despite pervasive global efforts to prevent and treat postpartum hemorrhage via expanded access to uterotonic drugs [18], particularly injectable oxytocin and ergometrine which are heat-labile, similar efforts have not been targeted toward uterotonic drug quality management [8].

Until recently, most studies of uterotonic drug potency dated from the 1990s. In 1993, a WHO simulation study testing the stability of injectable uterotonic drugs showed that after 12 months oxytocin lost no active ingredient under refrigeration (4°–8° Celsius). Three-to-seven percent active ingredient was lost after 12 months at 21°–25° Celsius, and 9%–19% active ingredient was lost after 12 months at 30° Celsius. Oxytocin was not affected by exposure to light. Ergometrine was shown to be much less stable. Ergometrine lost 5% of its active ingredient after 12 months at 4°–8° Celsius (in darkness). When exposed to light, it lost more than 90% of its active ingredient when stored for 12 months at 21°–25° Celsius [19]. A few other studies were identified that assessed degradation of oral and injectable uterotonics from environmental exposure. In general, these studies supported the findings from the WHO simulation that ergometrine was much less stable under tropical conditions than oxytocin, and that both posed public health concerns in contexts without access to refrigeration [20–22].

In 2010, a uterotonic drug quality study was conducted in three contrasting regions of Ghana in which research assistants simulated clients, visited randomly selected pharmacies, and requested to purchase two ampoules of either oxytocin or ergometrine. All purchased ampoules (n =101) were then sent for chemical assay to determine the percent of active pharmaceutical ingredient (API) in each ampoule. Results showed that 76% of the oxytocin ampoules and 100% of the ergometrine ampoules were outside manufacturer specification for API [23]. Post-marketing surveillance which resulted from this study found 39 of 40 (97.5%) and 38 of 40 (94.9%) injectable oxytocin and ergometrine samples, respectively, failed the assay for API or a sterility test. Ninety percent of the 118 ergometrine samples assessed in this surveillance exercise were imported from India [24].

No published studies of uterotonic drug quality from India were identified. In 2008, Bate and colleagues assessed the concentration of active ingredient in 541 units of ciprofloxacin, chloroquine, erythromycin, isoniazid and rifampicin in pharmacies in New Delhi and Chennai and found that 12% and 5% in each city, respectively, failed the test [9]. Seear and colleagues tested units of purchased rifampicin, artesunate and ciprofloxacin from pharmacies in Chennai, India in 2010 and found 44%, 80% and 6%, respectively, of units to be outside of manufacturer specification for API [25]; however, they found no evidence of counterfeiting and concluded that the poor quality drugs were likely due to poor manufacturing or decomposition during storage. Bate and colleagues cite a 2003 report by the Indian government which estimated that approximately 9% of all drugs sold at pharmacies in India were of substandard quality [9].

A small unpublished study by Peter Hall at the Concept Foundation of the quality of misoprostol tablets conducted in eight low and middle income countries including India, found problems with the content and purity of misoprostol from some manufacturers. In 34 of 76 samples tested across all eight countries, the active pharmaceutical ingredient was less than 90% of the labeled content. A key finding from this study is that appropriate packaging with aluminum over and under the misoprostol tablets (versus use of aluminum and plastic) is critical to its stability by preventing exposure to humidity during storage of the drug.

This study was designed as a sister-study to the Ghana uterotonic drug quality study in order to provide an assessment of the quality of oxytocin and ergometrine in two diverse world regions. This study has two objectives. The first objective is to assess the accessibility of four drugs commonly used for uterotonic purposes from a sample of private pharmacies in four districts in India. The four drugs are: oxytocin, methylergometrine, misoprostol and valethamate bromide (brand name: Epidocin). Valethamate bromide is not a uterotonic drug. It is a smooth muscle relaxant which is commonly used in India to relax the cervix and hasten delivery [26,27]. Here we refer to it as a uterotonic. Unlike the other drugs studied here, in this paper we refer to valethamate bromide by its brand name which is highly recognized in contrast to its nonproprietary name. The second objective is to assess the potency of ampoules of oxytocin and methylergometrine purchased by simulated clients. For this objective, we focus only on uterotonics known to be in common use during labor and the immediate postpartum period in India.

## Methods

To increase generalizability of results, two states (Karnataka and Uttar Pradesh) with contrasting socio-economic indicators were selected. Within each of these states, two districts (Hassan and Bagalkot in Karnataka; Agra and Gorakhpur in Uttar Pradesh) with contrasting maternal health indicators were selected. Socio-economic and maternal health indicators in Karnataka greatly surpass those in Uttar Pradesh (Table 1). For example, literacy among women of reproductive age and household electricity are substantially higher and fertility is substantially lower in Karnataka than in Uttar Pradesh [28]. The population of Uttar Pradesh state is more than three times larger than that of Karnataka (199.6 million vs 61.1 million in 2011), and Agra and Gorakhpur combined are 2.4 times larger than Bagalkot and Hassan [29,30]. Hassan and Gorakhpur are the more rural districts of Karnataka and Uttar Pradesh, respectively. Antenatal care and medical assistance at birth are more than twice as high in Karnataka than Uttar Pradesh [31].

**Table 1:** **Socio-economic and maternal health indicators, by state and district**

**State-level indicators**				
**State**	**Population in 000’s in 2011**	**% of women who are literate**	**% of households with electricity**	**Total fertility rate**
Karnataka	61130	60.3	89.0	2.1
Uttar Pradesh	199581	43.0	43.0	3.8
**District-level indicators**				
	**Population in '000 s in 2011**	**% rural**	**% of births with ≥3 antenatal care visits**	**% of births with medically trained attendant**
**Karnataka state**				
Bagalkot	1890	71.0	63.0	75.6
Hassan	1776	82.3	94.0	99.1
**Uttar Pradesh state**				
Agra	4380	56.7	20.5	44.9
Gorakhpur	4436	80.4	43.3	43.6

Sources: [30–33].

The sampling plan for this study was nested within the design of a parallel study in which a representative sample of public health facility-based deliveries was selected and observed, with an emphasis on documenting uterotonic drug use before and after delivery of the baby and other related behaviors by the birth attendant [34]. For the observation of deliveries study, the sampling frame was restricted to public health facilities with at least 72 deliveries per year, which eliminated 80 of the 203 facilities identified. Sixteen public health facilities in each district were selected with probability proportional to their annual volume of deliveries, for a total of 64 public health facilities. The sampling frame for this uterotonic drug accessibility and potency study includes all private pharmacies surrounding those 64 public health facilities, as pharmacies tend to cluster around health facilities in India. The decision to incorporate the sample design of the uterotonic drug accessibility/potency study into the observation of deliveries study was made for two reasons: 1) women delivering in public facilities in India and in many low income countries are asked to purchase uterotonics at private pharmacies when facility stocks are low; and 2) private pharmacies are a likely source of uterotonics for women delivering at home, where unmonitored use of these drugs is common [32,33].

The size of this study was driven by the amount of funding available to test oxytocin and methylergometrine; we gave each drug equal importance, and decided to split the funds equally between the two, leading to a maximum of approximately 200 assays for each drug, across all the districts. This number was sufficient to estimate the proportion of samples within API bounds with approximately 4%-7% precision, depending on the underlying true potency rate (i.e. 90% to 50%, respectively). For analysis purposes, two ampoules purchased from the same pharmacy is considered as one unit for analysis. The purchase of two ampoules per pharmacy was done to accommodate possible breakage or the need to retest. To maximize the sample available for subsequent within-district estimation of the private sector accessibility of drugs, we decided to achieve our per-drug pharmacy-visit target of 200 by allocating an equal number of pharmacies per district (50) to each drug. This sample size allows for construction of 95% confidence intervals of the overall, state, and district-specific estimates of drug accessibility of maximum width 7%, 10%, and 14% respectively (i.e. if true accessibility is ~50%).

To compile the sampling frame of private pharmacies, four teams of two research assistants and one field supervisor set out on foot to list the name and address of all pharmacies within one or five kilometers of selected health facilities. The size of the study area surrounding each health facility varied by the number of pharmacies identified; in areas where fewer than five pharmacies were found within one kilometer of the health facility, research assistants expanded the perimeter of their search to five kilometers. Once the sampling frame was completed, study staff at headquarters then selected pharmacies for the purchase of oxytocin by choosing a random start number and systematically selecting pharmacies with a constant sampling interval until reaching a sample of 50 pharmacies. The three consecutive pharmacies following those selected for the purchase of oxytocin were selected for the purchase of methylergometrine, misoprostol and Epidocin, respectively.

Field staff were trained for 2.5 days to compile the sampling frame of pharmacies, act as simulated clients, document their purchases and assure that cold chain conditions were maintained for the storage and transport of the purchased drugs. All field staff were male. Trainings were conducted in Lucknow, Uttar Pradesh and Bangalore, Karnataka. Staff from the Social Research Institute, India, collaborated with the authors on training and data collection. The sampling frame was compiled during the month of July 2011.

Beginning in August 2011 in Uttar Pradesh and September 2011 in Karnataka, research assistants visited their assigned pharmacies; each asked to purchase the assigned drug for his sister or wife who was soon to deliver. Research assistants assigned to purchase oxytocin, methylergometrine or Epidocin attempted to purchase two ampoules per visit. Those assigned to misoprostol attempted to purchase three tablets, the recommended dosage for PPH prevention. When requested, simulated clients provided an informal prescription which consisted of a handwritten note bearing the logo of a commonly used analgesic drug. Informal prescriptions were prepared for human subjects purposes to avoid putting the salesperson in the position of being asked to sell drugs without a prescription even after he/she had asked for one.

Shortly after leaving the pharmacy, research assistants placed purchased ampoules or tablets in plastic bags with coded labeling to identify the district, pharmacy, drug purchased, form of the drug (ampoule or tablet) and number of ampoules or tablets purchased. The plastic bags were then placed in vaccine cold chain carriers until the evening, at which point they were placed in refrigerators, generally at the local health facility. Within six weeks of data collection, all ampoules of oxytocin and methylergometrine were transported in the cold chain to VIMTA laboratory, a WHO pre-qualified laboratory, in Hyderabad, India. VIMTA documented that all ampoules were delivered under cold-chain conditions.

Ampoules of oxytocin and methylergometrine were refrigerated until analysis at which point they were assayed for the percent of active pharmaceutical ingredient (API). Analysis was conducted following the Finished Pharmaceutical Product specifications of the United States Pharmacopeia (USP) monograph #33 for both drugs. The USP chemical reference standard (Batch No.: F2K133, Cat. No.: 1491300) was used for oxytocin and the European Pharmacopeia Chemical reference standard (Lot No.: 1, Cat. No.: Y000076) was used for the analysis of methylergometrine. Ampoules were tested without blinding to product packaging, as this information is required for testing. Due to requirements for ethical approval of the study, manufacturer names are not reported.

Accessibility was estimated as the proportion of visits to open pharmacies that resulted in a successful purchase of the target drug. Similarly, prescription requirement was estimated as the proportion of visits to open pharmacies for which purchase of the target drug required a prescription. Both proportions and corresponding exact binomial confidence intervals were estimated separately for each drug and district. For each of the assayed drugs (oxytocin and methylergometrine), the relationship between API and the time elapsed between manufacture and purchase was initially assessed by examining locally weighted scatterplot smoothing. As these plots indicated an approximately linear relationship, both simple linear regression and cubic spline models were estimated. Analysis of residuals was utilized to assess for influential outliers, and after the removal of two points, the predicted API from the final models were plotted against the actual values. The absolute loss of API per 90 days elapsed was estimated, along with a 95% confidence interval. Stata (Version 11) software was used for data analysis. To note, we assume no further loss of API from the point of data collection to assay given that the purchased drugs were stored in the cold chain during this time.

### Ethics statement

There was no verbal or written informed consent for this study, as consent of the salesperson would have undermined the simulated client methodology. The study protocol was approved by the following boards: Clinicom, the state-level ethics committee in Karnataka; Chhatrapati Shahuji Maharaj Medical University; Uttar Pradesh Ethics Committee in Lucknow, Uttar Pradesh; the Health Ministry Screening Committee and the Indian Council on Medical Research in New Delhi; the PATH Research Ethics Committee in Seattle, Washington, USA; and the Johns Hopkins Bloomberg School of Public Health Institutional Review Board in Baltimore, Maryland, USA. In addition, the study was approved by the Directorates of Health and Family Welfare in Karnataka and Uttar Pradesh.

## Results

### Accessibility of uterotonic drugs

A total of 877 pharmacies were visited by simulated clients across all four districts; 200 each in Agra and Gorakhpur, Uttar Pradesh (as planned), 244 in Bagalkot and 233 in Hassan, Karnataka. A total of 77 additional pharmacies were selected after the beginning of fieldwork in Bagalkot and Hassan due to the lower than expected availability of oxytocin and methylergometrine there. All selected pharmacies in Agra and Gorakhpur were open and visited by simulated clients. In Bagalkot and Hassan, 14 and 16 of the selected pharmacies, respectively, were closed during the teams’ visits. In 847 pharmacies simulated clients were able to speak with a salesperson.

Two aspects of accessibility of uterotonic drugs are explored: the simulated client’s success at purchasing the assigned drug and need for a prescription. Success at purchasing assigned drugs varied significantly by state, but in general, not between districts within the same state. For example, in Agra and Gorakhpur, Uttar Pradesh, 90%-100% of visits for each of the four drugs resulted in a purchase. In Bagalkot and Hassan, Karnataka, only 29%-52% of visits for each drug resulted in a purchase and oxytocin and methylergometrine were successfully purchased in only about one-third of visits. This may imply a general lack of uterotonics in stock in private pharmacies, low availability of these drugs in pharmacies given their common availability in hospitals or suspicion by the sales person leading to reluctance to sell to the simulated client in Karnataka. The only significant difference in accessibility of uterotonics between districts in Karnataka was for Epidocin (Table 2). The need for informal prescriptions varied by drug and by district (Table 3). Informal prescriptions were requested in less than 10% of pharmacy visits in Bagalkot and Hassan, Karnataka. In Agra, informal prescriptions were rarely requested except for oxytocin. In Gorakhpur, informal prescriptions were requested from 8%-44% of pharmacy visits depending on the assigned drug; except for oxytocin, the need for a prescription was higher in this district compared to the other three. In only seven pharmacy visits (six in Hassan and one in Bagalkot) was a prescription requested but no sale made. No additional comments were provided by the simulated clients to suggest that the cause was the informal prescription.

**Table 2:** **Percent (and exact binomial confidence intervals) of open private pharmacies at which a successful purchase was made by simulated clients by assigned uterotonic drug and district**

	**Bagalkot, Karnataka**			**Hassan, Karnataka**			**Agra, Uttar Pradesh**			**Gorakhpur, Uttar Pradesh**		
	**P**	**N**	**% and 95% CI**	**P**	**N**	**% and 95% CI**	**P**	**N**	**% and 95% CI**	**P**	**N**	**% and 95% CI**
Oxytocin	24	62	38.7 (26.6 – 51.9)	24	61	39.3 (27.1 – 52.7)	45	50	90.0 (78.1 – 96.7)	50	50	100 (92.9 – 100)*
Methylergometrine	23	77	30.0 (20.0 – 41.3)	23	69	33.3 (22.4 – 45.7)	50	50	100 (92.9 – 100)*	48	50	96.0 (86.3 – 99.5)
Misprosotol	16	43	37.2 (23.0 – 53.3)	23	44	52.3 (36.7 – 67.5)	48	50	96.0 (86.3 – 99.5)	48	50	96.0 (86.3 – 99.5)
Epidocin	24	46	52.2 (36.9 – 67.1)	13	45	28.9 (16.4 – 44.4)	50	50	100 (92.9 – 100)*	49	50	98.0 (89.4 – 99.9)
Open private pharmacies		228			219			200			200	

*one-sided 97.5% confidence interval.

**Table 3:** **Percent (and exact binomial confidence intervals) of attempts to purchase uterotonic drugs in which a prescription was requested by assigned drug and district**

	**Bagalkot, Karnataka**			**Hassan, Karnataka**			**Agra, Uttar Pradesh**			**Gorakhpur, Uttar Pradesh**		
	**P**	**N**	**% and 95% CI**	**P**	**N**	**% and 95% CI**	**P**	**N**	**% and 95% CI**	**P**	**N**	**% and 95% CI**
Oxytocin	0	62	0 (0 – 5.8)*	3	61	4.9 (1.0 – 13.7)	10	50	20.0 (10.0 – 33.7)	4	50	8.0 (2.2 – 19.2)
Methylergometrine	3	77	3.9 (1.0 – 11.0)	4	69	5.8 (1.6 – 14.2)	2	50	4.0 (0.5 – 13.7)	11	50	22.0 (11.5 – 36.0)
Misprosotol	1	43	2.3 (0.1 – 12.3)	4	44	9.1 (2.5 – 21.7)	1	50	2.0 (0.1 – 10.6)	22	50	44.0 (30.0 – 58.7)
Epidocin	1	46	2.2 (0.1 – 11.5)	3	45	6.7 (1.4 – 18.3)	0	50	0 (0 – 5.8)*	13	50	26.0 (14.6 – 40.3)

*one-sided 97.5% confidence interval.

### Drug potency

Table 4 presents the number of ampoules received at the laboratory and analyzed by drug. A total of 193 ampoules of oxytocin and 188 ampoules of methylergometrine were received at the laboratory for testing. Three ampoules of oxytocin in Hassan and four ampoules of methylergometrine were reported purchased but not received at the laboratory. Ampoules of oxytocin received at the laboratory were produced by 17 different manufacturers, nine in Karnataka and eight in Uttar Pradesh. For methylergometrine, parallel numbers are two and three manufacturers, respectively. Although the study design required two ampoules per pharmacy purchase as a unit of analysis, the number of successful purchases in Bagalkot and Hassan, Karnataka led to the decision to test all ampoules from these districts. In Uttar Pradesh, only one ampoule per sample was tested for API. The second ampoule, where there was one, was held as a back-up in case of breakage; 20 ampoules of both oxytocin and methylergometrine came from sites that provided only one ampoule per pharmacy in Uttar Pradesh.

**Table 4:** **Number of oxytocin and methylergometrine ampoules received and analyzed for chemical testing, by district**

	**Number of oxytocin ampoules received and analyzed**	**Number of methyl-ergometrine ampoules received and analyzed**
**State/District**		
**Karnataka state**		
Bagalkot	48 (2 each from 24 sites; all analyzed)	42 (2 each from 23 sites, missing 4; all analyzed,)
Hassan	45 (2 each from 24 sites, missing 3; all analyzed,)	46 (2 each from 23 sites; all analyzed)
**Uttar Pradesh state**		
Agra	50 (1 each from 36 sites providing pairs, 14 from sites providing 1 ampoule only; all analyzed)	50 (1 each from 46 sites providing pairs, 4 from sites providing 1 ampoule only; all analyzed)
Gorakhpur	50 (1 each from 40 sites providing pairs, 10 from sites providing 1 ampoule only; all analyzed)	50 (1 each from 40 sites providing pairs, 10 from sites providing 1 ampoule only; all analyzed)
Total number of ampoules analyzed	193	188

Note: All ampoules received at the laboratory from Karnataka state were analyzed. In Uttar Pradesh, in sites where two ampoules were provided per pharmacy, laboratory staff randomly selected one for chemical testing.

The percent distribution of the API in ampoules of oxytocin and methylergometrine are presented by district in Table 5. The percent of oxytocin ampoules outside manufacturer specifications (90%-110% API) ranged from 33%-40% in Karnataka and from 22%-50% in Uttar Pradesh. Small percentages of oxytocin ampoules showed API from 110%-120%, and less than 11% of the ampoules within any district showed oxytocin at 50% API or less. The median API for ampoules of oxytocin across all four districts ranges from 98.5%-102.9%. None of the ampoules of oxytocin were expired at the time of testing.

**Table 5:** **Percent distribution of Active Pharmaceutical Ingredient (API) in purchased ampoules of oxytocin and methylergometrine, by district**

	**Karnataka state**				**Uttar Pradesh state**			
	**Bagalkot**		**Hassan**		**Agra**		**Gorakhpur**	
**% API**	**OXYTOCIN (%)**	**METHYL-ERGOMETRINE (%)**	**OXYTOCIN (%)**	**METHYL-ERGOMETRINE (%)**	**OXYTOCIN (%)**	**METHYL- ERGOMETRINE (%)**	**OXYTOCIN (%)**	**METHYL- ERGOMETRINE (%)**
0%-25%	0.0	0.0	8.9	0.0	2.0	2.0	0.0	2.0
26-50%	0.0	9.5	2.2	0.0	6.0	2.0	0.0	0.0
51%-75%	4.2	7.1	4.4	13.0	14.0	8.0	2.0	6.0
76%-89%	27.1	83.3	22.2	82.6	16.0	42.0	10.0	36.0
WITHIN MANU-FACTURER SPECIFICA-TION (90%-110%)	66.7	0.0	60.0	4.3	50.0	46.0	78.0	56.0
111%-120%	2.1	0.0	2.2	0.0	12.0	0.0	8.0	0.0
Missing data	0.0	0.0	0.0	0.0	0.0	0.0	2.0	0.0
TOTAL	100.0	100.0	100.0	100.0	100.0	100.0	100.0	100.0
% Out of specification	33.0	100.0	40.0	95.7	50.0	54.0	22.0	44.0
Median API	98.5	84.4	98.7	85.8	99.0	89.2	102.9	90.4
N of ampoules of analysis	48	42	45	46	50	50	50	50

Results for the potency of methylergometrine were less favorable. In Bagalkot and Hassan, Karnataka 96% and 100% of the methylergometrine ampoules were outside manufacturer specification, respectively. In Agra and Gorakhpur, Uttar Pradesh 54% and 44% were outside manufacturer specification, respectively. Likewise, median API scores across all four districts were lower than those for oxytocin, ranging from 84.4%-90.4%. None of the methylergometrine ampoules were expired.

As mentioned above, the limited availability of oxytocin and methylergometrine in the study districts in Karnataka led to assaying all of the ampoules purchased there, in contrast to UP where one ampoule was assayed per pharmacy purchase in most cases. This design change had little effect on the percentage of ampoules reported as out of manufacturer specification, as the potency results are nearly identical when both ampoules from the same pharmacy are assayed. The absolute median difference in the percent API among pharmacy-pairs of purchased oxytocin and methylergometrine ampoules is 0.10% and 0.05%, respectively. This is not surprising as the ampoules were likely from the same batch and would have been exposed to the same environmental conditions. Of 46 pharmacy-pairs of oxytocin ampoules from Karnataka, in only five pairs was one within manufacturer specification and the other outside specification. All 44 pharmacy-pairs of methylergometrine ampoules were either both within or both outside manufacturer specification.

**Figure 1:**
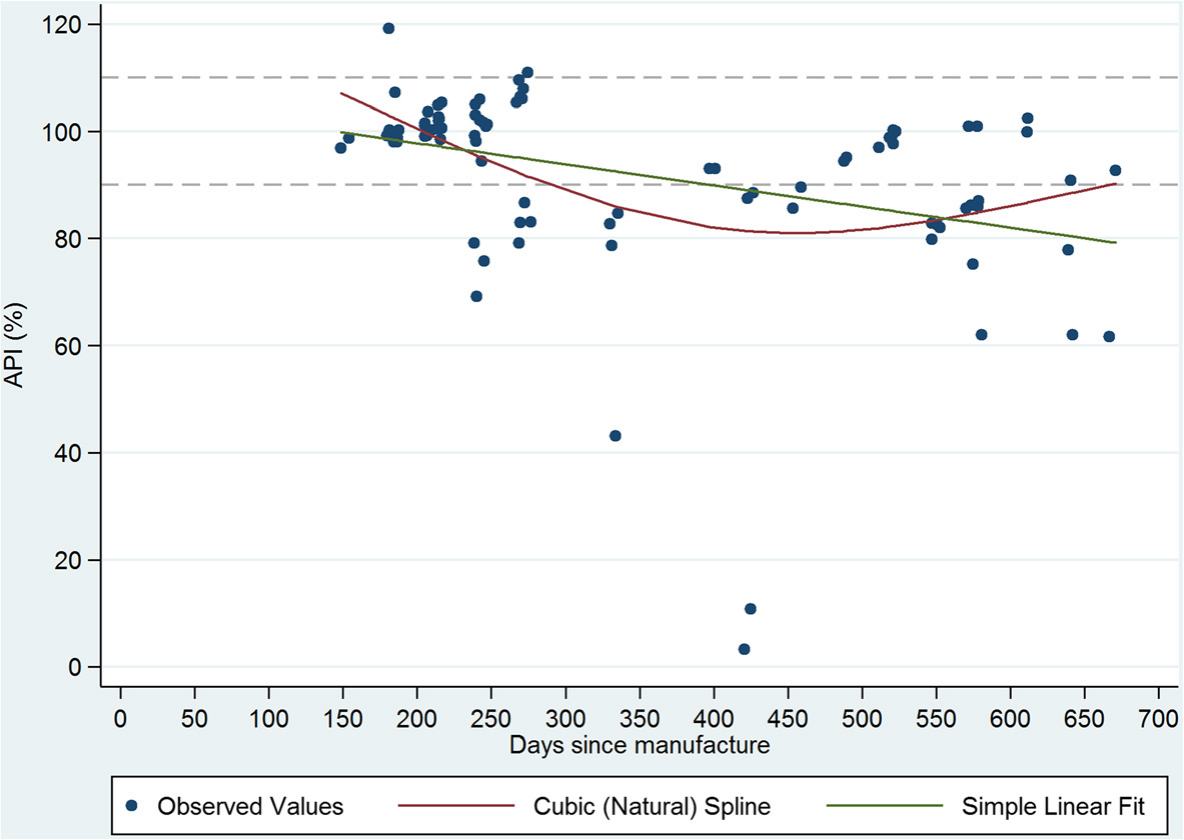
**Karnataka: Measured Active Pharmaceutical Ingredient (in %) by time between drug manufacture and purchase; oxytocin.**

**Figure 2:**
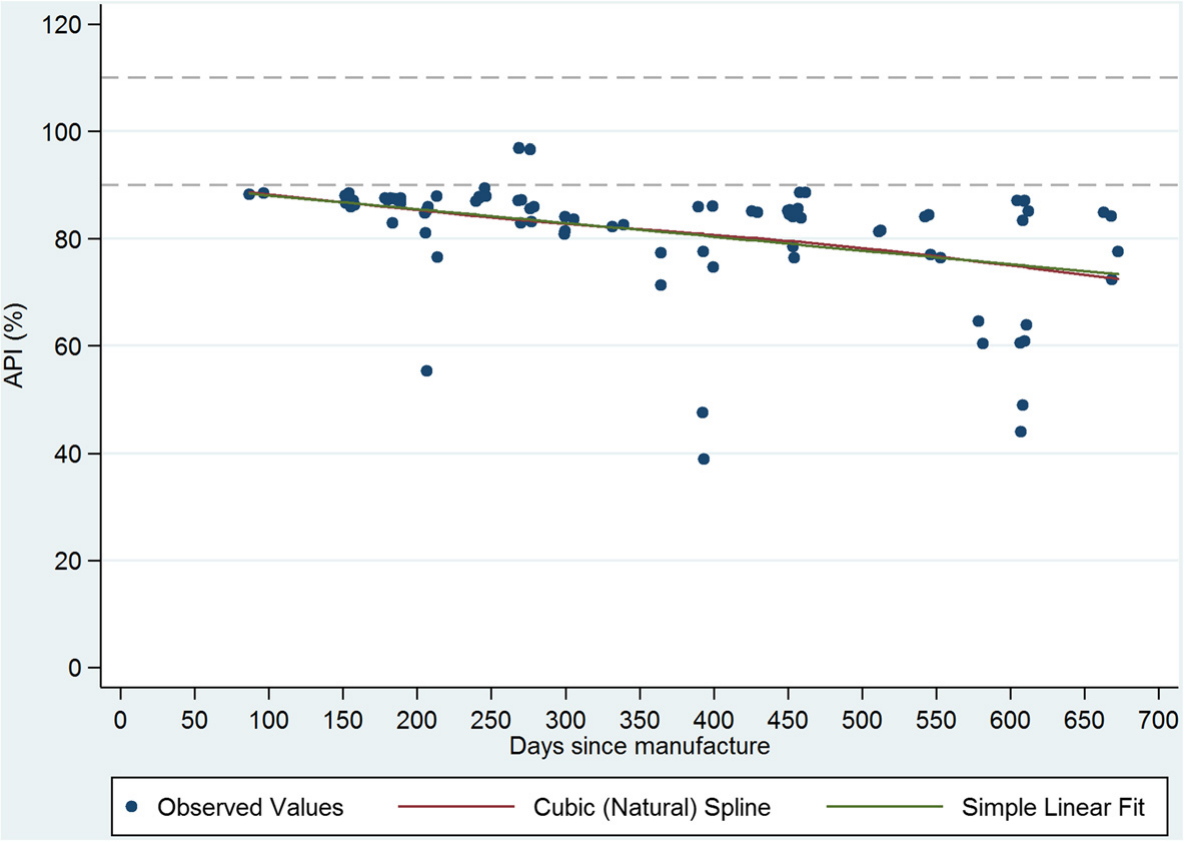
**Karnataka: Measured Active Pharmaceutical Ingredient (in %) by time between drug manufacture and purchase; methylergometrine.**

**Figure 3:**
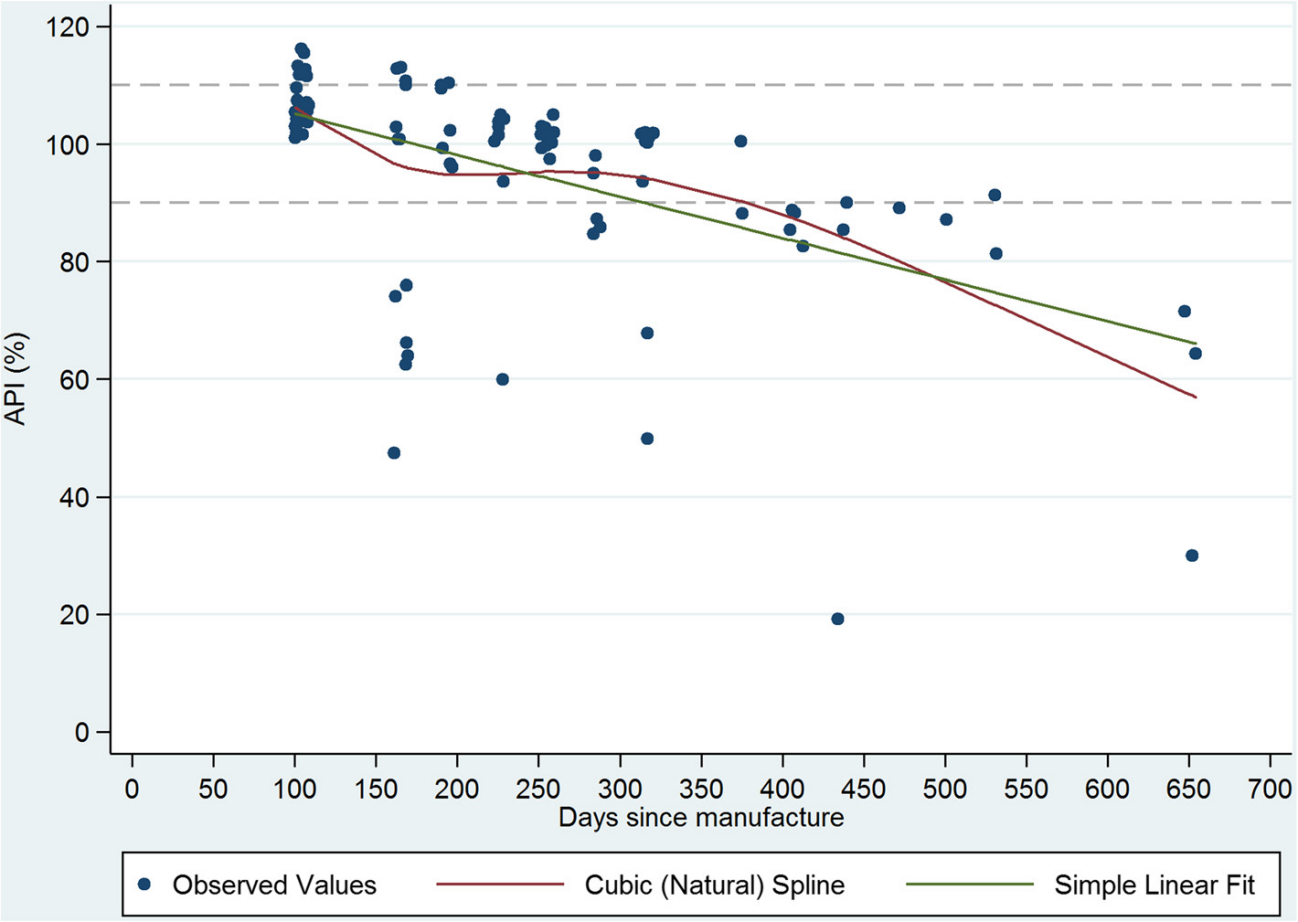
**Uttar Pradesh: Measured Active Pharmaceutical Ingredient (in %) by time between drug manufacture and purchase; oxytocin.**

**Figure 4:**
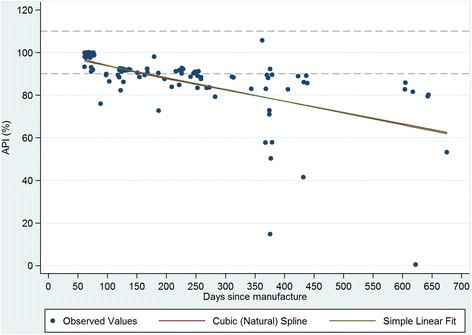
**Uttar Pradesh: Measured Active Pharmaceutical Ingredient (in %) by time between drug manufacture and purchase; methylergometrine.**

Figures 1, 2, 3 and 4 plot the percent API for oxytocin and methylergometrine by state and by time between dates of manufacture and purchase of ampoules. The negative slope of the regression lines for both drugs in both states is supportive, but not proof, of environmental degradation as an explanation for the API outside of manufacturer specification. In Karnataka, for oxytocin and methylergometrine, the approximate loss of API per 90 days was 3.6% (1.6%-5.5%) and 2.3% (1.1%-3.5%), respectively (Figures 1 and 2). In Uttar Pradesh, for oxytocin and methylergometrine, the approximate loss of API per 90 days was 6.4% (4.4%-8.4%) and 5.0% (3.5%-6.5%), respectively (Figures 3 and 4).

Simulated clients recorded where the purchased ampoules of oxytocin and methylergometrine were kept in each pharmacy. See Table 6. In the districts in Uttar Pradesh, oxytocin was kept unrefrigerated on the shelf in 93% (Agra) and 100% (Gorakhpur) of pharmacies in which oxytocin units were purchased. In the districts in Karnataka, this practice is much less common, with around 15% of pharmacies placing purchased oxytocin ampoules unrefrigerated on the shelf, although not all purchased ampoules were in view of simulated clients. For methylergometrine, 83% of pharmacies kept methylergometrine unrefrigerated on the shelf for purchase in Gorakhpur, as did at least two-thirds of the pharmacies in Agra.

**Table 6:** **Percent distribution of the placement of oxytocin and methylergometrine in pharmacies where these drugs were purchased, by district**

	**Oxytocin**				**Methylergometrine**			
**State/District**	**On shelf (%)**	**Refrigerated (%)**	**Could not see (%)**	**N of pharmacies where oxytocin was purchased**	**On shelf (%)**	**Refrigerated (%)**	**Could not see (%)**	**N of pharmacies where methyl-ergometrine was purchased**
**Karnataka state**								
Bagalkot	16.7	83.3	0.0	24	8.7	91.3	0	23
Hassan	12.5	79.2	8.3	24	30.4	39.1	30.4	23
**Uttar Pradesh state**								
Agra	93.3	2.2	4.4	45	66.0	16.0	18.0	50
Gorakhpur	100.0	0.0	0.0	50	83.3	16.7	0.0	48
Total N				143				144

## Discussion

This study sought to assess accessibility of drugs commonly used for uterotonic purposes in India and to assay the potency of oxytocin and methylergometrine available in private pharmacies. In Uttar Pradesh, the simulated clients documented easy access to all four study drugs. In Karnataka, accessibility was much lower, implying a possible lack of uterotonics in these districts, though we do not know if such shortages are frequent. Given the small percentage of pharmacies requesting a prescription in both states and the fact that informal prescriptions used for this study sufficed 99% of the time, it appears that weak enforcement of regulations for prescription drugs permits easy access to uterotonic drugs in this population.The potency of oxytocin and methylergometrine varied by state, district and drug. However, even the best-case scenario (in Gorakhpur, Uttar Pradesh) suggests that one in five ampoules of oxytocin available to the population in private pharmacies may be outside manufacturer specifications for API. The worst-case scenario for oxytocin was shown in Agra where one in two ampoules of oxytocin was out of specification. Serious problems were documented in the two study districts in Karnataka where 96%-100% of methylergometrine ampoules tested were outside manufacturer specification for API. This is of particular concern as methylergometrine is often used for PPH treatment due to the tetanic contractions it produces. However, although API fell outside quality thresholds for some proportion of both drugs, fewer than 10% of the ampoules tested at 50% API or less. None of the ampoules showed 0% API (one indicator of a possible counterfeit drug) and none were expired at the time of testing. No data are available to relate the documented losses of API shown here for either drug to clinical outcomes, though it is unlikely that any Ministry of Health would endorse use of drugs out of manufacturer specification. All results were shared with the Ministry of Family Health and Welfare at the national and state levels.

The strengths of this study include an up-to-date sampling frame of private pharmacies compiled for the study and a sample of oxytocin and methylergometrine that is likely free of bias thanks to the simulated client methodology which prevents pharmacy staff from influencing the selection of ampoules for assay. In addition, the similarity of results regarding drug quality from four contrasting sites suggests that uterotonic drug quality is likely to be an issue in other districts of Karnataka and Uttar Pradesh, and in other states in India. The MEDQUARG checklist for field studies of medicine quality developed by Newton and colleagues [13] was used in designing and reporting on this study and is recommended by these authors.

There are two limitations to this study. The first is that sample size for potency testing was based on constraints regarding the cost of the chemical assays. There is no consensus on sample design for drug quality studies [13,25]; detailed data on the volume and the flow of drugs through private supply chains are difficult to obtain, particularly in a country the size of India. In this study in four districts, 17 different local manufacturers of oxytocin were identified. However, these constraints still provided us with high a-priori expected precision of estimated API (i.e. 4% to 7% precision). The second limitation is that interpretation of the drug potency results requires caution. One cannot say why the drugs were out of specification for API. Their potency at manufacture, at departure from the manufacturer, or along the supply chain is unknown. Although exposure to high temperatures (oxytocin, methylergometrine) and light (methylergometrine) likely contributed to the low APIs documented here, it is curious that drug potency for methylergometrine in Karnataka is worse than in Uttar Pradesh given that higher percentages of these uterotonics were stored outside refrigeration in Uttar Pradesh than in Karnataka. On the other hand, the relationship shown in Figures 1, 2, 3 and 4 suggesting an approximate loss of API per 90 days ranging from 3.6% to 6.4% for oxytocin and from 2.3% to 5.0% for methylergometrine, is highly suggestive of drug degradation. The results of the assays for both drugs are not suggestive of counterfeit drugs.

It is surprising that private-sector accessibility of uterotonic drugs and the potency of private-sector methylergometrine were worse in Karnataka than in Uttar Pradesh given the relatively advantaged status of Karnataka state. Likewise, superior indicators in Gorakhpur relative to Agra were not anticipated.

## Conclusion

These results underscore the necessity of routine drug surveillance and monitored pharmaceutical storage conditions in the private sector. The status of uterotonic drugs in the government health system is unknown. As a result of this study, the protocol for a follow on study assessing uterotonic drug potency in public health facilities and the supply chain is currently underway in Karnataka and Uttar Pradesh.

Development of a mini-lab kit for field-based assessment of oxytocin, similar to kits in current use for antimicrobial, antiretroviral, anti-tuberculosis and antimalarial drugs [35], is urgently needed. Given that methylergometrine is currently listed as a second-line drug for postpartum hemorrhage prevention and treatment by WHO [3], and that this is the second recent study to show the large majority if not the entire sample of ergometrine/methylergometrine ampoules outside of specification [23], a broader discussion regarding the replacement of methylergometrine/ergometrine with another drug or immediate attention to storage conditions for this drug is warranted. Should newer uterotonic drugs such as carbetocin and carbopost become commonly available in India, an expanded assessment of uterotonic drugs will also be warranted.

## Abbreviations

API: Active pharmaceutical ingredient
PPH: Postpartum hemorrhage
WHO: World Health Organization
